# Supplementary material for: Development of intravenously administered synthetic RNA virus immunotherapy for the treatment of cancer
Source: Nat Commun. 2022 Oct 7;13:5907. doi: 10.1038/s41467-022-33599-w (PMC9546900; doi:10.1038/s41467-022-33599-w)
Supplement: Supplementary file 1 — Supplementary Information [file 41467_2022_33599_MOESM1_ESM.pdf]

Supplementary Information

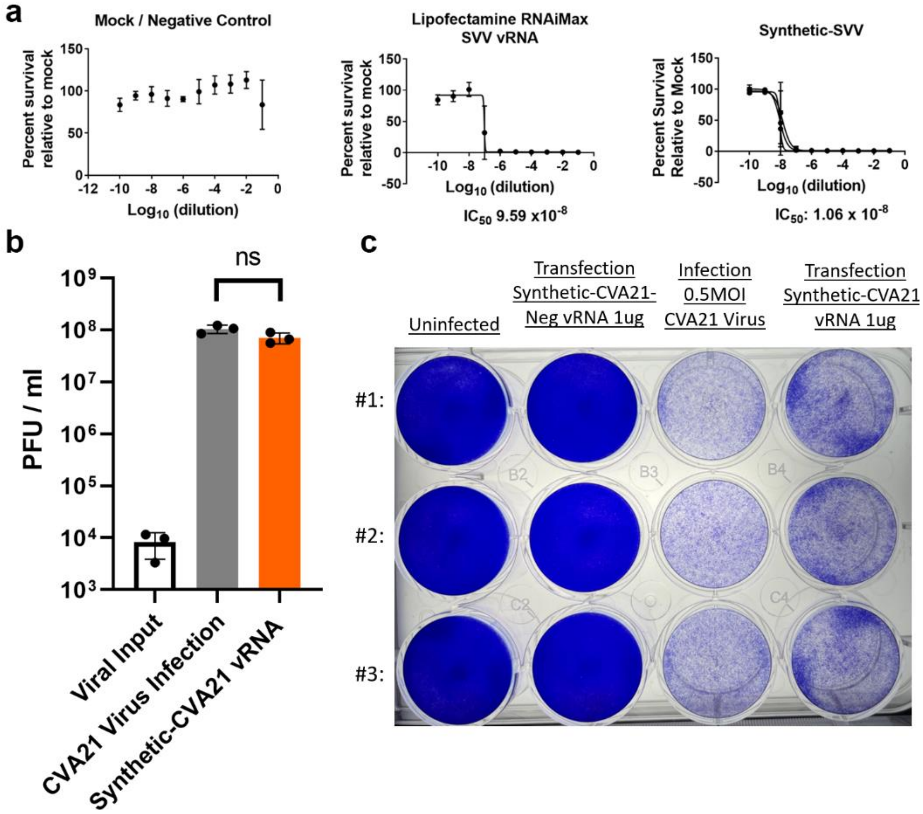

Supplementary Figure 1: vRNA transfection produces infectious virus. (**a**) NCI-H1299 cells were transfected with 1 µg of SVV-Neg RNA (negative control) or 1 µg SVV-001 RNA or 1 µg of LNP formulated Synthetic-SVV (SVV-001 vRNA). After 2 days, supernatants were filtered through 0.45 µm PVDF filters, and infectious virions were quantified by IC_{50} on permissive NCI-H446 cells, n=3 per dilution, means and SD are shown. (**b-c**) NCI-H1299 cells were transfected with1 µg CVA21-Neg RNA, 1 µg CVA21 RNA, or infected with CVA21 virus. (**b**) After 72 hrs, infectious virions in the supernatant were quantified by plaque titer. Input virus was assessed by plaque titer of supernatant recovered from wells collected immediately after adding CVA21 at MOI of 0.5, n=3. Data are presented as mean values +/- SD. An unpaired two tailed students T test was applied. ns, P = 0.18 (**c**) To illustrate cell lysis, 72h post-infection/transfection wells were stained with crystal violet, n=3.

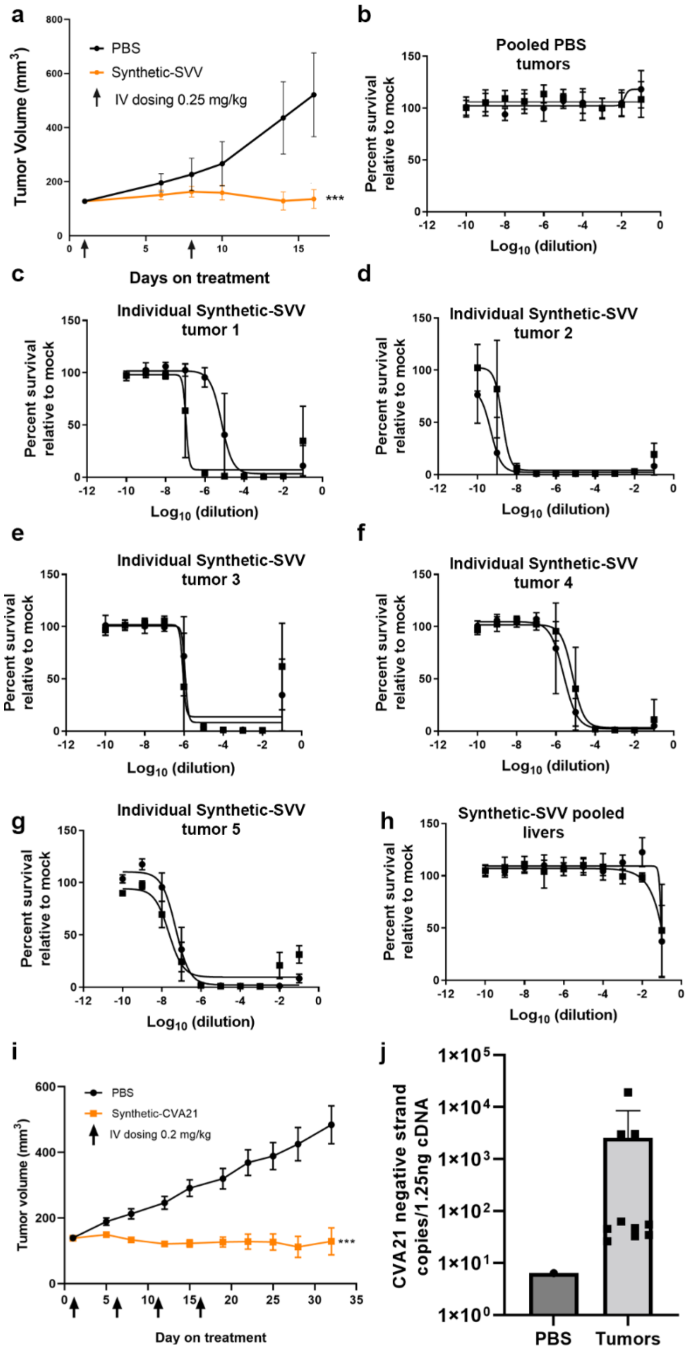

Supplementary Figure 2: Synthetic virus induces tumor growth inhibition and generates virions in tumors. (**a**) Athymic nude mice (n = 6 per group) were implanted subcutaneously with NCI-H1299 NSCLC xenograft tumors. Mice were treated by IV administration with either PBS vehicle control or Synthetic-SVV 0.25 mg/kg (SVV-001) on Days 1 and 8. Tumor volume (mm^3) was monitored at various time points. Data are presented as mean values +/- SEM. Statistical significance was determined using a mixed linear model *** $p < 0.001$. (**b-h**) Tumors and liver were collected on Day 16. Cleared tissue lysates were prepared, and the presence of SVV-virions was assessed in an IC_{50} assay. Data are presented as mean values +/- SD. (**b**)

Cleared tumor lysates from PBS mice were pooled. (**c-g**) Individual (n = 5) cleared tumor lysate from mice treated with Synthetic-SVV. (**h**) Cleared liver lysates from mice treated with Synthetic-SVV were pooled (n = 5). (**i-j**) Athymic nude mice (n = 10 per group) were implanted with SK-MEL-28 human melanoma tumors subcutaneously. Mice were treated by IV administration with either PBS vehicle control or Synthetic-CVA21 0.2 mg/kg on Days 1, 6, 11, and 16. (**i**) Tumor volume (mm^3) was monitored at various time points. Data are presented as mean values +/- SEM. Statistical significance was determined using a mixed linear model *** $p < 0.001$. (**j**) At the end of the study (Day 32), tumors were collected. CVA21 negative-strand RNA levels were determined via RT-qPCR. Data are presented as mean values +/- SD.

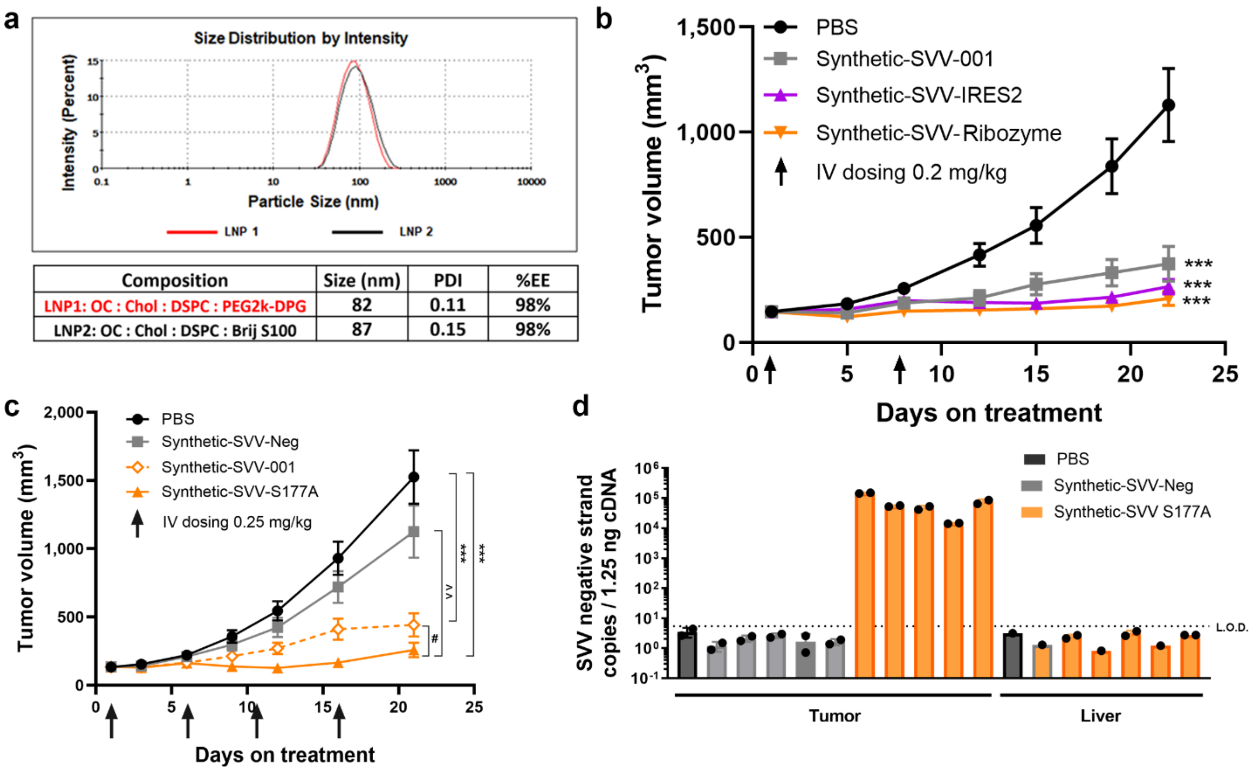

Composition	Size (nm)	PDI	%EE
LNP1: OC : Chol : DSPC : PEG2k-DPG	82	0.11	98%
LNP2: OC : Chol : DSPC : Brij S100	87	0.15	98%

Supplementary Figure 3: Optimal vRNA design and LNP formulation result in improved efficacy. (**a**) Dynamic light scattering results are shown for 2 representative formulations whose compositions are depicted in the lower panel table. (**b-d**) Athymic nude mice (n = 10 per group) were implanted subcutaneously with NCI-H1299 NSCLC xenograft tumors. (**b**) Mice were treated by IV administration with either PBS vehicle control or Synthetic-SVV 0.2 mg/kg (SVV-001, SVV-IRES2, SVV-Ribozyme) on Days 1 and 8. Tumor volume (mm^3) was monitored at various time points. Data are reported as mean ± s.e.m. Statistical significance was determined using a mixed linear model *** $p < 0.001$ vs. PBS. (**c-d**) Mice were treated by IV administration with either vehicle control (PBS), Synthetic-SVV-Neg, or Synthetic-SVV 0.25 mg/kg (SVV-001- SVV-S177A) on Days 1, 6, 11, and 16. (**c**) Tumor volume (mm^3) was monitored at various time points. Data are reported as mean ± s.e.m. Statistical significance was determined using a mixed linear model, *** $p < 0.001$ vs. PBS; ^^ $p < 0.01$ vs. Synthetic-SVV-Neg, and # $p < 0.05$ vs. Synthetic-SVV-001. (**d**) At the end of the study (Day 21), tumors and livers were collected. SVV negative-strand RNA levels were determined via RT-qPCR (n = 5 per time point). The RT-qPCR limit of detection (LoD) is denoted with a dotted line. Data are presented as mean values.

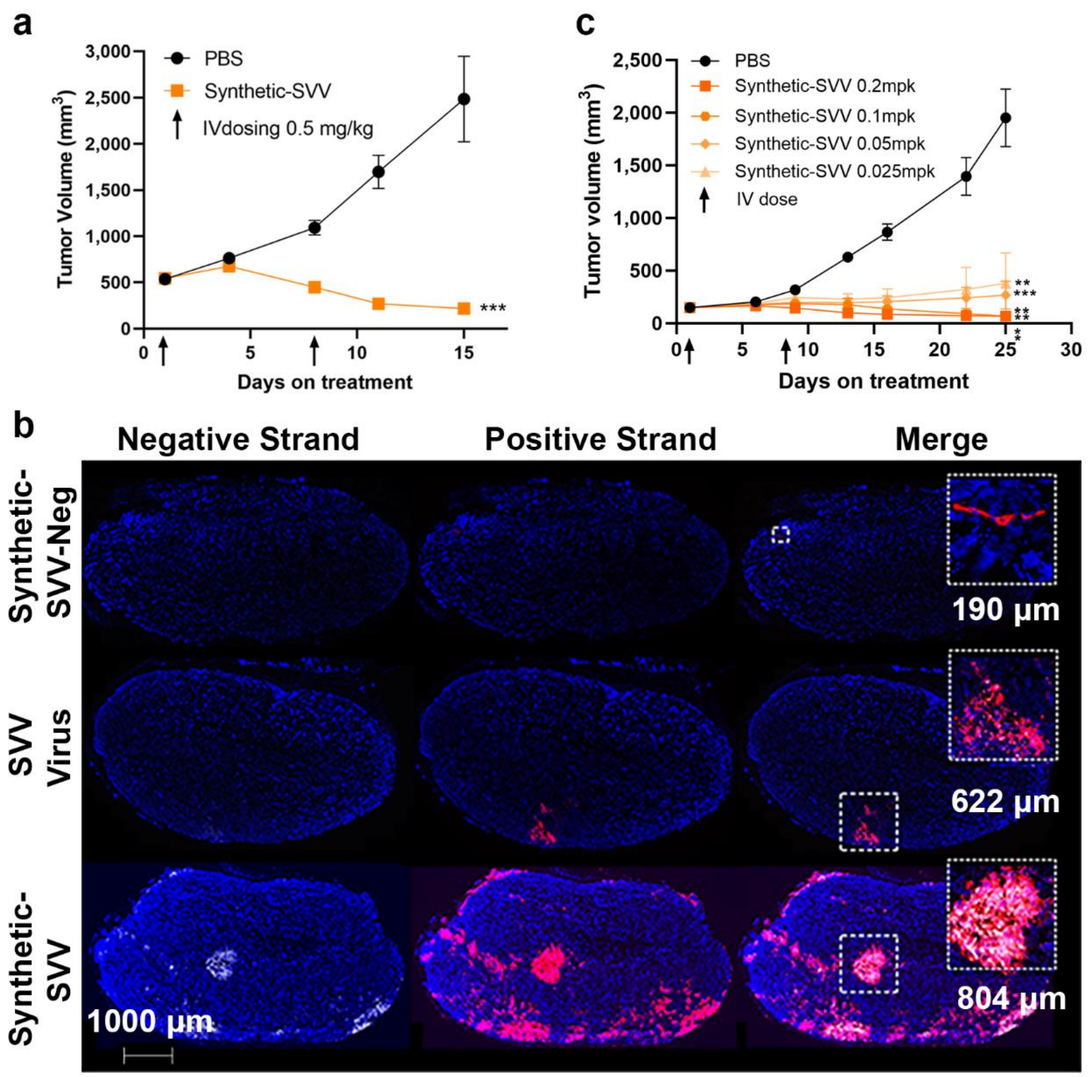

Supplementary Figure 4: Synthetic-SVV is efficacious against large SCLC tumors. (**a-c**) Athymic nude mice were implanted subcutaneously with NCI-H446 SCLC xenograft tumors. **(a)** Mice were treated (n = 8 per group) by IV administration with either PBS vehicle control or Synthetic-SVV 0.5 mg/kg on Days 1 and 8. Treatment was initiated when the average tumor volume reached 550 mm^3. Tumor volume (mm^3) was monitored at various time points. Data are reported as mean ± s.e.m. Statistical significance was determined using a mixed linear model *** $p < 0.001$ vs. PBS. **(b)** FISH specific for the SVV positive (red) or the negative (white) RNA strands are shown for a section of whole NCI-H446 xenograft tumors treated with one dose (1 mg/kg) of Synthetic-SVV-Neg, SVV virus (10^6 PFU), or Synthetic-SVV and collected after 72 hr. Nuclei were labelled with 4',6-diamidino-2-phenylindole (DAPI). Insets: high magnification images depicted to scale by the white box; scale measurement included below. These images are representative of n=4 individual tumor images. **(c)** Mice (n = 7 per group) were treated by IV administration with either PBS vehicle control or Synthetic-SVV on Days 1 and 8 at doses ranging from 0.025 to 0.2 mg/kg. Tumor volume (mm^3) was monitored. Data are reported as mean ± s.e.m. Statistical significance was determined using a mixed linear model ** $p < 0.01$ and *** $p < 0.001$ vs. PBS.

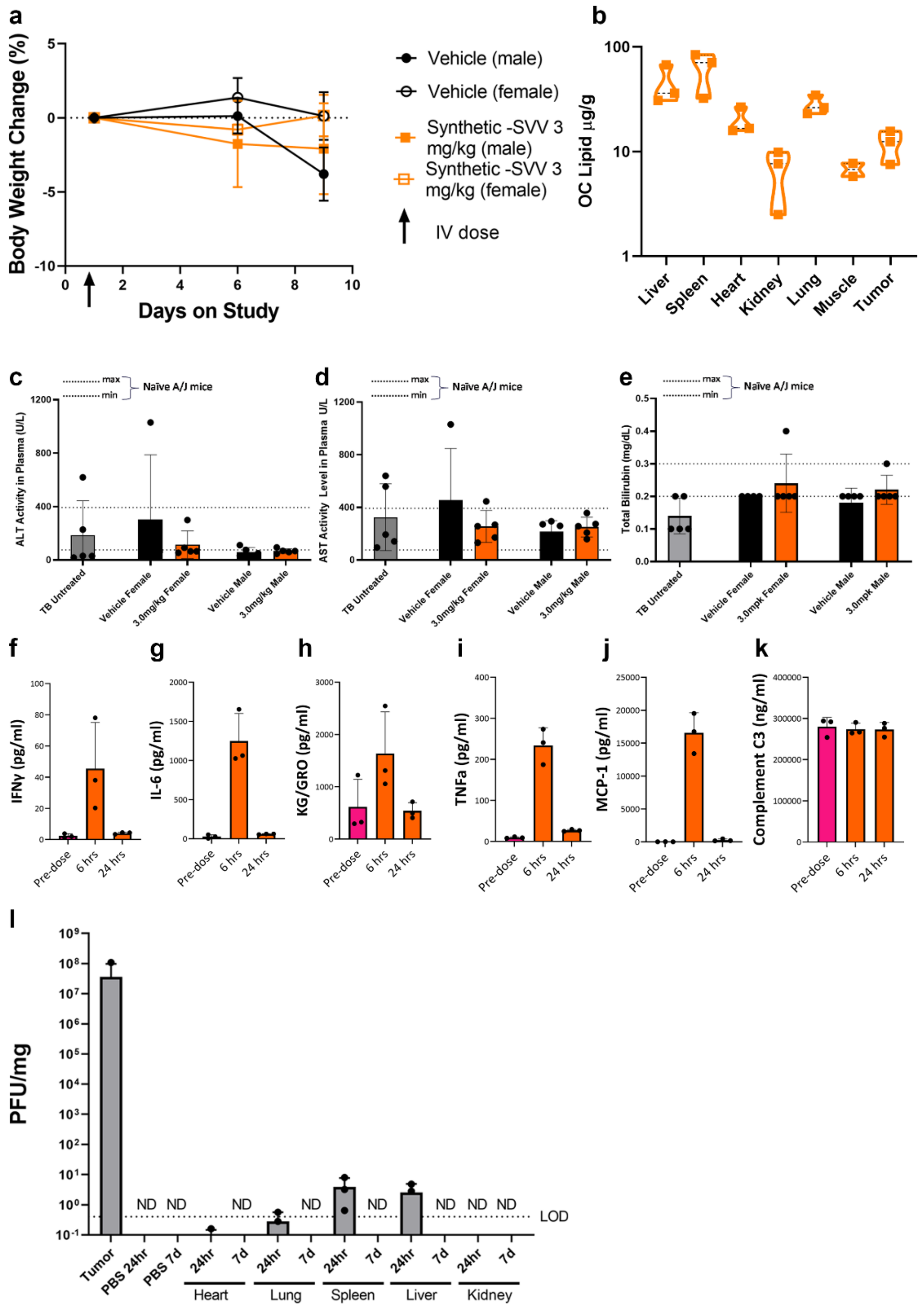

Supplementary Figure 5: Synthetic-SVV is well tolerated in immuno-competent A/J mice. **(a-l)** Tolerability assessment of systemic administration of Synthetic-SVV at 3 mg/kg in permissive A/J mice bearing N1E-115 neuroblastoma tumor model (n = 4 per group). Data are presented as mean values +/- SEM. (**a**) Changes in body weight of mice on treatment. (**b**) Ionizable lipid (OC) biodistribution was quantified by LC-MS and normalized to tissue weight in

tissue samples collected after 30 min post-dose. (**c-e**) Liver chemistry in plasma 24 hr post-dose. (**c**) Alanine aminotransferase (ALT) activity. (**d**) Aspartate aminotransferase (AST) activity. (**e**) Total bilirubin. (**f-j**). Plasma cytokine and chemokines levels in mice pre-dose and 6 and 24 hr post-dose (**f**) Interferon-gamma (IFNγ). (**g**) Interleukin-6 (IL-6). (**h**) Keratinocyte chemoattractant (KC)/human growth-regulated oncogene (GRO). (**i**) Tumor necrosis factor-alpha (TNF-α). (**j**) Monocyte chemoattractant protein-1 (MCP-1). (**k**) Complement C3 levels in mice pre-dose and 6 and 24 hr post-dose. (**l**) A/J mice (n = 3 per group) were dosed with Synthetic-SVV at 3 mg/kg. Tissues were collected after 24 hr and 7-days post-dosing, and plaque titer assays for SVV were conducted from tissue homogenates. Positive control sample is derived from Synthetic-SVV treated tumor. Limit of detection (LoD) is denoted with a dotted line. (b-l) Data are presented as mean values +/- SD.

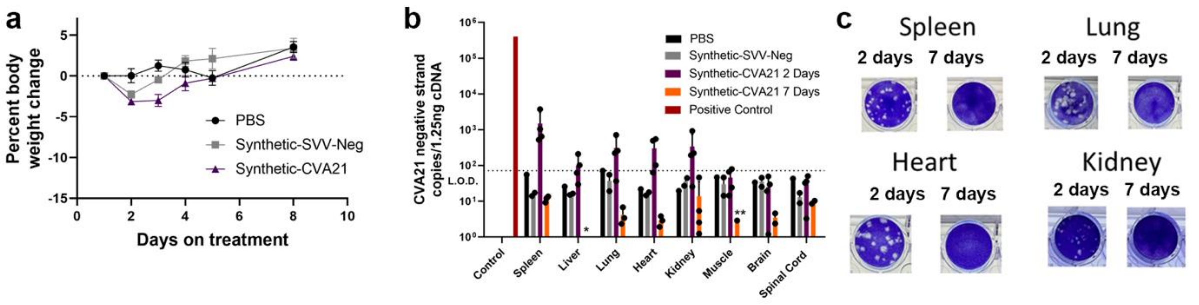

Supplementary Figure 6: CVA21 is rapidly cleared from hICAM1 transgenic mouse tissues after administration of Synthetic-CVA21. Tolerability assessment of systemic administration of Synthetic-CVA21 at 1.6 mg/kg in permissive huICAM1 transgenic mice (n = 4 per group). Data are presented as mean values +/- SEM. (**a**) Changes in body weight of mice on treatment. (**b-c**) Tissues were collected after 2- and 7-days post-dosing. RT-qPCR and plaque titer assay for CVA21 were conducted from tissue homogenates. (**b**) Viral replication in tissues was assessed by RT-qPCR of the CVA21 negative-strand RNA; * and ** indicate samples had replicates with no detectable signal. The positive control sample is derived from CVA21 infected NCI-H1299 cells in culture. The RT-qPCR limit of detection (LoD) is denoted with a dotted line. Data are presented as mean values +/- SD (**c**) Representative plaque titer results are shown from whole tissue homogenates.

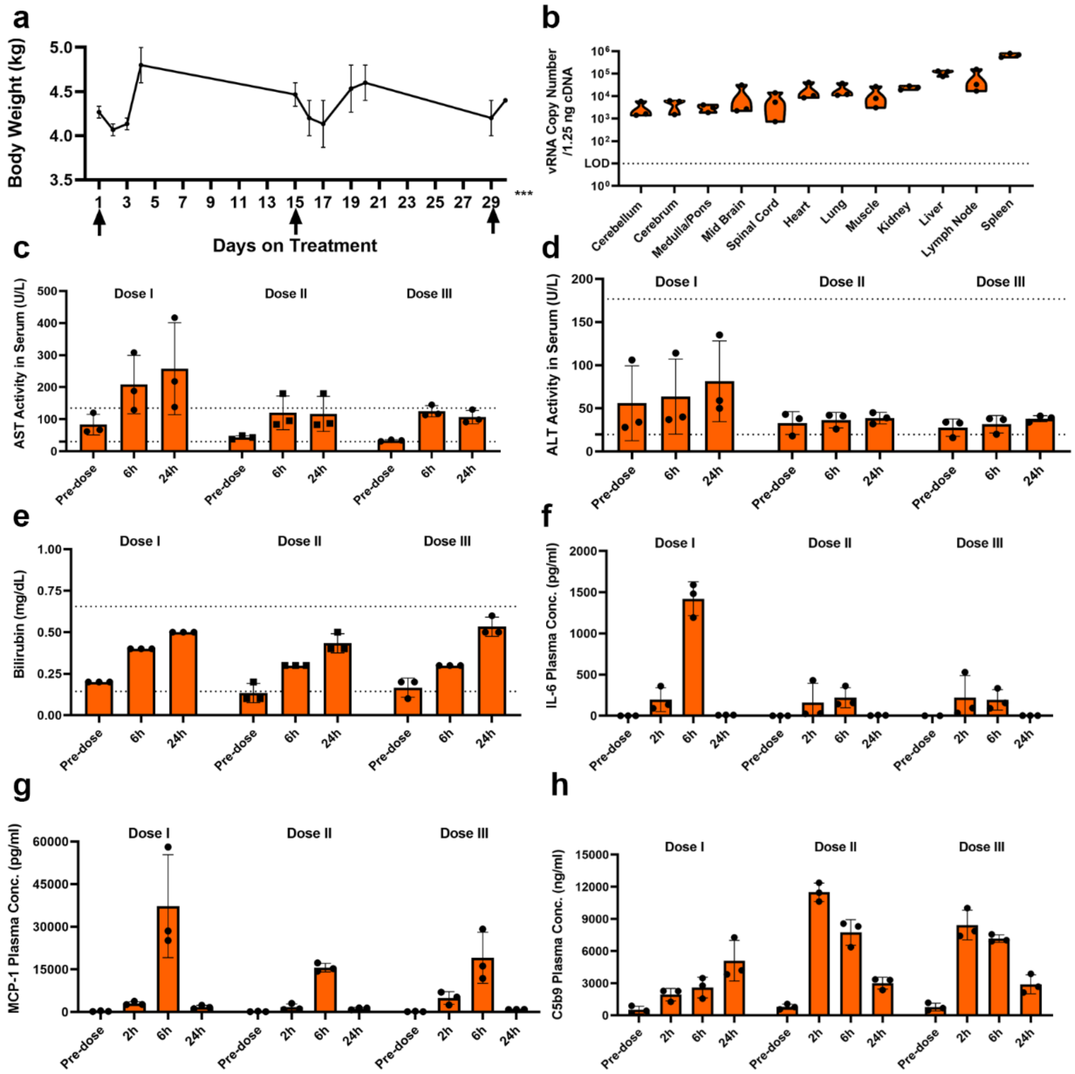

Supplementary Figure 7: Synthetic-SVV is well tolerated in cynomolgus monkeys. (a-h) Tolerability and biodistribution assessment of systemic administration of Synthetic-SVV (three doses at 1 mg/kg every 2 weeks) male cynomolgus monkeys (n = 3). (**a**) Body weight of monkeys on treatment. Data are presented as mean values +/- SEM. (**b**) RT-qPCR analysis of positive-strand SVV RNA in tissues 24 hr following third IV dose of Synthetic-SVV **(c-e)** Clinical chemistry analysis at pre-dose, 6 and 24 hours following each treatment: **(c)** aspartate aminotransferase (ALT) activity, **(d)** alanine aminotransferase (ALT) activity; **(e)** bilirubin plasma concentration. (**f-g**) Plasma cytokine and chemokines levels in monkeys at pre-dose 2, 6, and 24 h post-dose **(f)** Interleukin-6 (IL-6). **(g)** Monocyte chemoattractant protein-1 (MCP-1). (**h**) Complement C5b9 levels in monkeys at pre-dose 2, 6, and 24 h post-dose. **(b-h)** Data are presented as mean values +/- SD. The dotted lines represent normal ranges of these parameters.

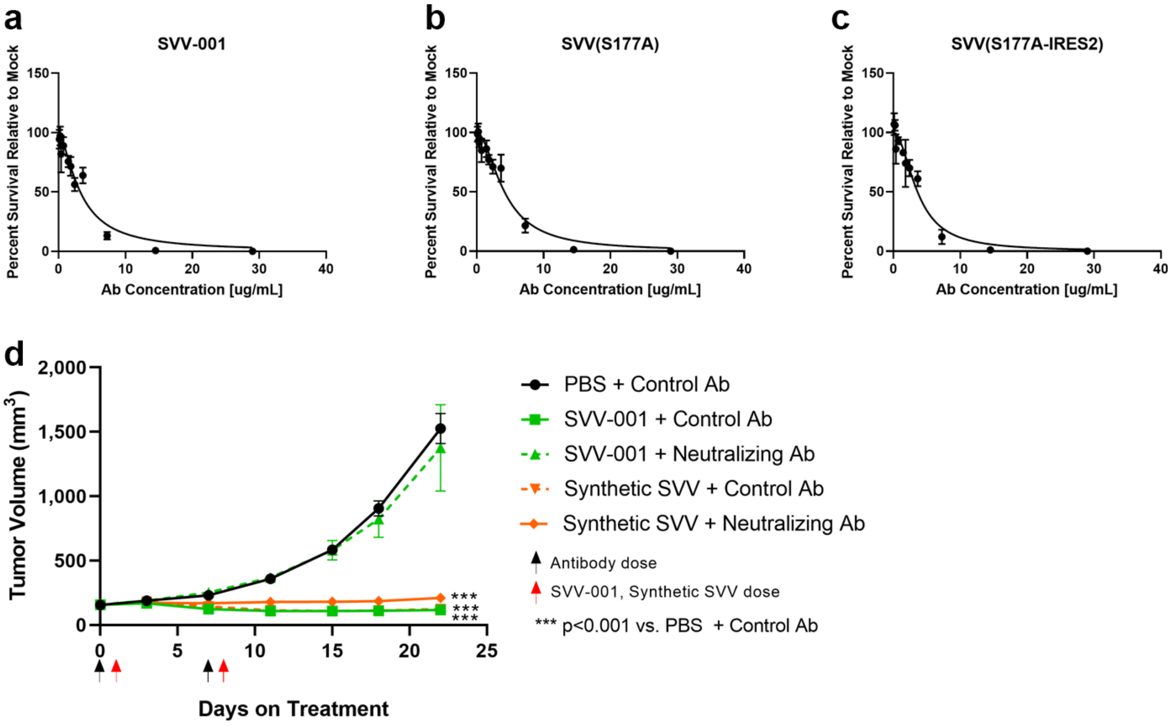

Supplementary Figure 8: Characterization of the neutralization potency of anti-SVV rabbit polyclonal. (a-c) SVV viral neutralization assay was tested in the SVV permissive NCI-H446 cell line. 1 x 10^6 $TCID_{50}$ of SVV-virions (SVV-001, SVV(S177A), and SVV(S177A-IRES2) encoded by Synthetic-SVV) were neutralized after incubation with a 1:320 dilution of an anti-SVV polyclonal rabbit serum (29 mg/mL) raised against UV inactivated SVV-001. n=3 replicates per dilution were performed **(d)** Anti-tumor efficacy of SVV-virions and Synthetic-SVV as assessed by tumor volume (mm^3) was evaluated in NCI-H446 tumor bearing mice after injection of control or anti-SVV rabbit serum. Mice passively immunized with SVV antisera received 2 IV injections of either 10^6 PFU of SVV-001 virions or 1.0 mg/kg Synthetic-SVV (n = 10 per group). Data is reported as mean ± s.e.m. Statistical significance was determined using a mixed linear model *** $p < 0.001$ vs. PBS + Control Ab.

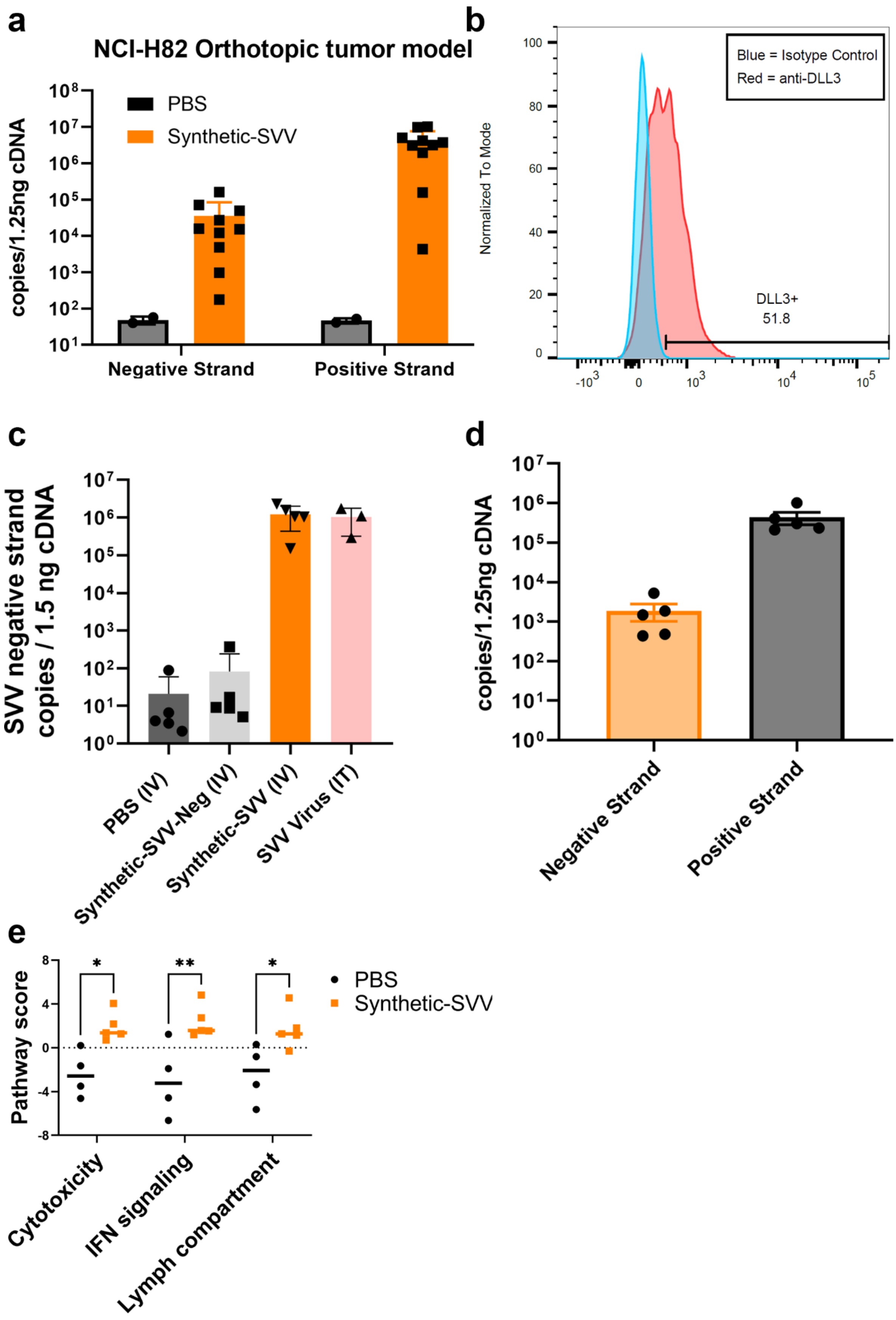
a
NCI-H82 Orthotopic tumor model
copies/1.25ng cDNA
PBS
Synthetic-SVV
Negative Strand
Positive Strand
b
Blue = Isotype Control
Red = anti-DLL3
Normalized To Mode
DLL3+
51.8
c
SVV negative strand copies / 1.5 ng cDNA
PBS (IV)
Synthetic-SVV-Neg (IV)
Synthetic-SVV (IV)
SVV Virus (IT)
d
copies/1.25ng cDNA
Negative Strand
Positive Strand
e
Pathway score
PBS
Synthetic-SVV
Cytotoxicity
IFN signaling
Lymph compartment

Supplementary Figure 9: Synthetic-SVV treatment leads to SVV viral replication and changes the tumor microenvironment in SCLC tumor models. (**a**) NCI-H82 tumors were orthotopically implanted in athymic nude mice. Animals were dosed either with PBS or 1.0 mg/kg Synthetic-SVV,15 days post-implantation (n = 10 per group). 96 hr post-dose, lungs were collected. SVV negative- and positive-strands RNA levels were determined via RT-qPCR. (**b**) Flow cytometric analysis of the expression of hDLL3 in NCI-H82 cells. Staining with specific anti-DLL3 antibody (pink shaded curve) or isotype-matched control (blue shaded curve). **(c)** NOD/SCID mice were implanted subcutaneously with SCLC PDX tumors (Crown Bioscience, San Diego, CA) and treated once by IV administration with either vehicle control (PBS), Synthetic-SVV-Neg, or Synthetic-SVV 1 mg/kg or twice intratumorally on Days 1 and 4 with 10^6 PFU SVV-virions. Tumor tissues were collected on Day 6 to determine viral replication. SVV negative-strand RNA levels were determined via RT-qPCR (n = 5 per group). **(d-e)** RPM mice were implanted subcutaneously with SCLC GEMM tumors and IV dosed with either vehicle control (PBS) or 1 mg/kg Synthetic-SVV on Day 1 and Day 8. Tumor tissues were collected 5 days post-second dose. **(d)** To determine viral replication, SVV negative-strand and positive-strand RNA levels were quantified by RT-qPCR in Synthetic-SVV treated group (n = 5). **(e)** mRNA was isolated from tumor tissues (PBS, n = 4; Synthetic-SVV, n = 5) and analyzed using the NanoString nCounter® PanCancer Mouse IO360. Statistical significance was determined using paired Two-way ANOVA p=0.02, p=0.004, p=0.03 from right to left **(a,c,d,e)** Data are presented as mean values +/- SD.

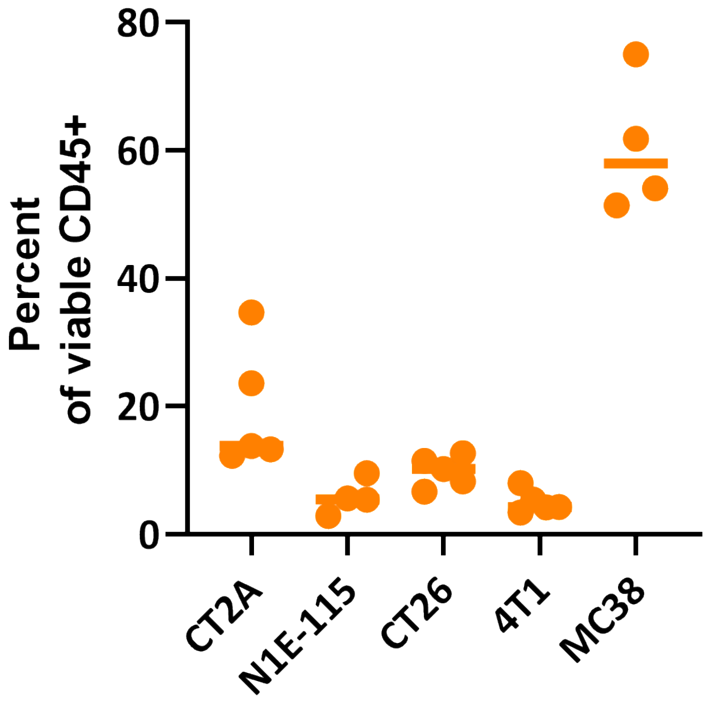

Supplementary Figure 10: N1E-115 tumors infiltrated with few immune cells. The immune cell infiltration in 5 different syngeneic models was assessed by flow cytometry in non-treated (PBS) mice 10 to 14 days after the tumors reached 150 mm^3 (n=5 per group). The panel shows the percentage of live CD45+ cells.

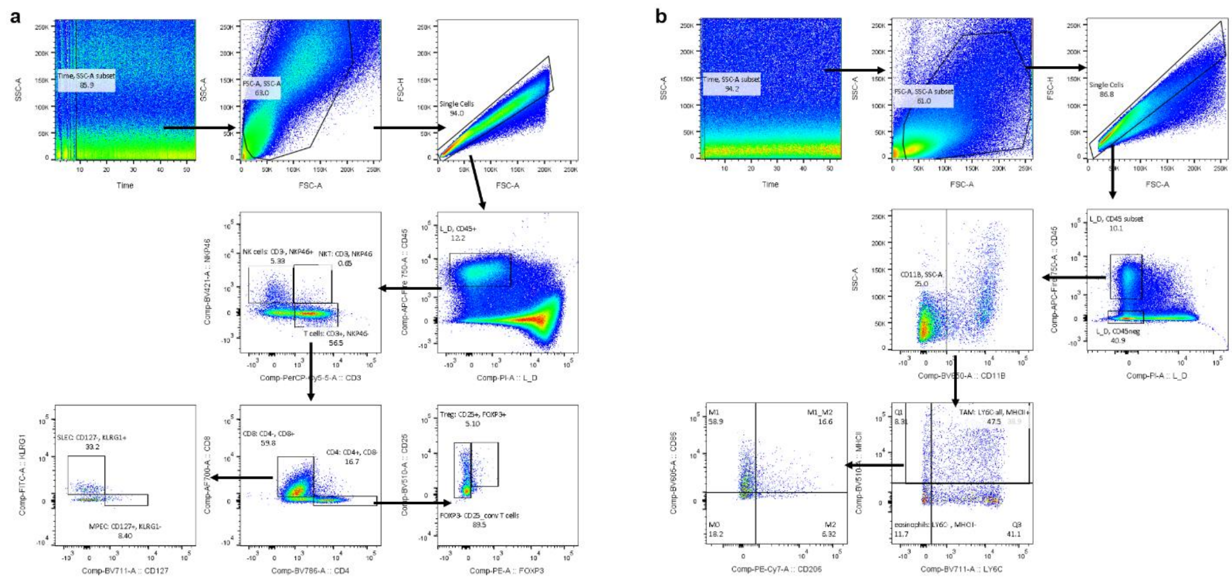

Supplementary Figure 11: Flow cytometry gating strategy for T cells, NK, and myeloid cells. (a) T cell and NK panel: cells were first gated for time (SSC-A vs. Time), lymphocytes (SSC-A vs. FSC-A), and singlets (FSC-H vs. FSC-A). The lymphocyte gate was further analyzed for their uptake of the Live/Dead stain to determine live versus dead cells and CD45 expression. Then, the cells were gated on CD3 versus NKP46 to select T cells or NK cells. For the T cells, the population was then gated for CD4 versus CD8. The CD4 T cells were further gated for CD25+ and FOXP3+ to analyze the Treg population, and CD8 T cells were further gated for KLRG1 and CD127 to assess their phenotype. NK cells were gated for NKP46+ and CD3-. Flow cytometry plots for the various markers are shown in bi-exponential format (logical plots). **(b)** Macrophage panel: cells were first gated for time (SSC-A vs. Time) then gated for lymphocytes (SSC-A vs. FSC-A) and singlets (FSC-H vs. FSCA). The lymphocyte gate was further analyzed for their uptake of the Live/Dead stain to determine live versus dead cells and CD45 expression. Then, macrophages were gated using SSC-A vs. CD11b+ and subsequently on MHCII+Ly6C- subset. To distinguish between M1 and M2 macrophages, we used CD206-CD86+ for M1 and CD206+CD86- for M2. Flow cytometry plots for the various markers are shown in bi-exponential format (logical plots).

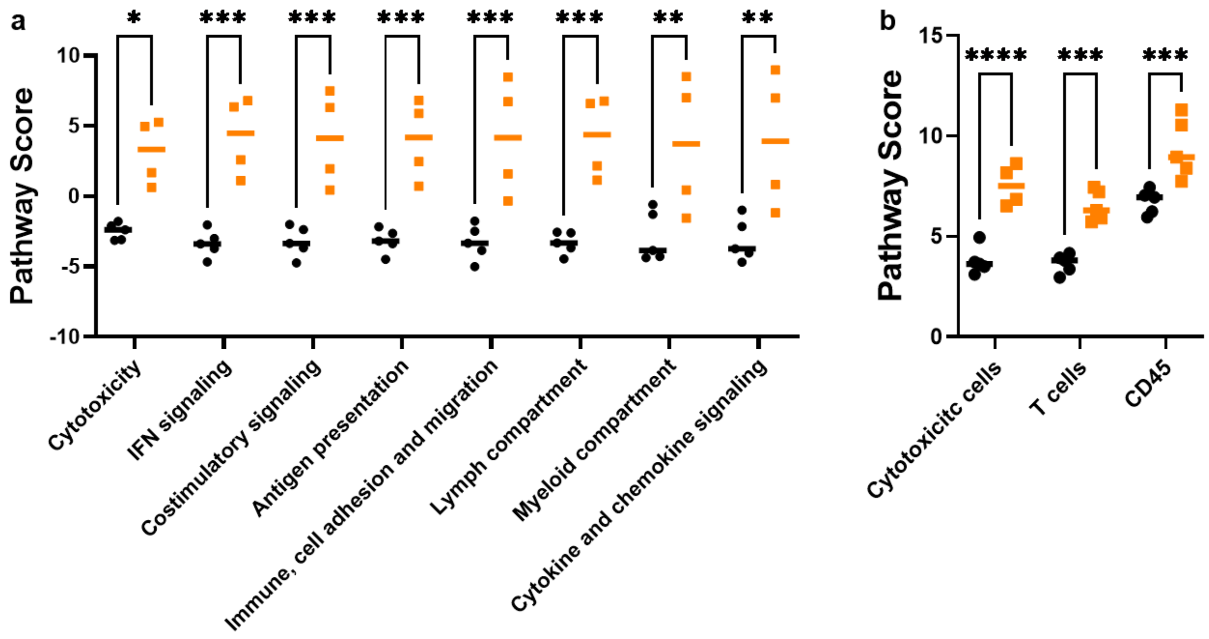

Supplementary Figure 12: Tumor Inflammation Signature. (a-b) A/J mice were implanted subcutaneously with N1E-115 tumor cells were IV dosed with either vehicle control (PBS, back circles) or 1 mg/kg Synthetic-SVV (orange squares) on Day 1 and Day 8. Tumor tissues were collected 5 days post-second dose. mRNA was isolated from tumor tissues (PBS, n = 5; Synthetic-SVV, n = 4) and analyzed using the NanoString nCounter ® PanCancer Mouse IO360. Data are presented as mean values +/- SD. **a)** Immune Pathway. From right to left p=0.01, 0.0003, 0.0006, 0.0008, 0.0005, 0.0004, 0.0029, 0.001 **(b)** Immune cells pathway. From right to left p>0.0001, 0.0001, 0.0003. Statistical significance was determined using paired Two-way ANOVA for all comparisons.

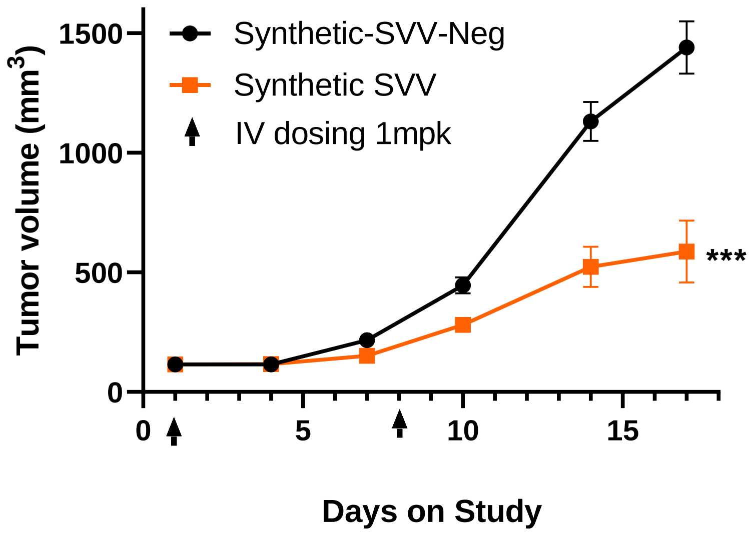

Supplementary Figure 13: Synthetic-SVV treatment is efficacious in a syngeneic neuroendocrine tumor model. A/J mice (n = 10 per group) implanted subcutaneously with syngeneic neuroendocrine N1E-115 tumors. Mice were treated by IV administration with either Synthetic SVV-Neg Synthetic-SVV at 1 mg/kg on Days 1 and 8. Tumor volume (mm^3) was monitored. Data are reported as mean ± s.e.m. Statistical significance was determined using a mixed linear model, *** $p < 0.001$ vs. Synthetic-SVV-Neg.

Supplementary Table 1: Pharmacokinetics of Synthetic-SVV in athymic nude mice and cynomolgus monkeys

Species	Lipid /(vRNA) Dose (mg/kg)*	N	Route	C_{max} (ug/ml)	$T_{½}$ (h)	AUC_{∞} (h*ug/ml)	V_{ss} (ml/kg)	CL (ml/h/kg)
**Athymic Nude Mouse**	**1.2/(0.1)**	3	IV bolus	13	0.8	12	140	104
**NHP**	**12/(1)**	3	IV infusion	229	3.6	983	82	12

***** Dose based on Ionizable lipid/(corresponding dose of vRNA).

Abbreviations: AUC, area under the curve; Cmax, maximum plasma concentration; CL, clearance; NHP, non-human primate; $T_{1/2}$, half-life;; V_{ss}, volume of distribution at steady-state; vRNA, viral RNA genome.

PK analysis of the individual plasma data was performed using Phoenix WinNonlin®, Version 8.3 (Certara, Princeton, NJ)). Kinetic parameters were estimated using a noncompartmental model (Plasma [200-202]), uniform weighting, IV dosing with sparse sampling, following linear-log trapezoidal calculation method).
