# Supplementary material for: Development of intravenously administered synthetic RNA virus immunotherapy for the treatment of cancer
Source: Nat Commun. 2022 Oct 7;13:5907. doi: 10.1038/s41467-022-33599-w (PMC9546900; doi:10.1038/s41467-022-33599-w)
Supplement: Supplementary file 3 — Description of Additional Supplementary Files [file 41467_2022_33599_MOESM3_ESM.pdf]

**Supplementary Data 1:** This file contains the raw Nanostring output associated with the data in Supplementary Figure 12; n=5 for vehicle (labeled NanostringVEHXXX) and n=4 for synthetic SVV (labeled NanostringSSVVXXX). It also includes the processed NanoString nCounter ® PanCancer Mouse IO360 data in the tab NanostringIO360scores.
