# Supplementary material for: Development of intravenously administered synthetic RNA virus immunotherapy for the treatment of cancer
Source: Nat Commun. 2022 Oct 7;13:5907. doi: 10.1038/s41467-022-33599-w (PMC9546900; doi:10.1038/s41467-022-33599-w)
Supplement: Supplementary file 5 — Reporting Summary [file 41467_2022_33599_MOESM5_ESM.pdf]

nature research

Corresponding author(s): Edward M. Kennedy

Last updated by author(s): Aug 29, 2022

Reporting Summary

Nature Research wishes to improve the reproducibility of the work that we publish. This form provides structure for consistency and transparency in reporting. For further information on Nature Research policies, see our Editorial Policies and the Editorial Policy Checklist.

Statistics

For all statistical analyses, confirm that the following items are present in the figure legend, table legend, main text, or Methods section.

n/a	Confirmed	
☐	☒	The exact sample size (n) for each experimental group/condition, given as a discrete number and unit of measurement
☐	☒	A statement on whether measurements were taken from distinct samples or whether the same sample was measured repeatedly
☐	☒	The statistical test(s) used AND whether they are one- or two-sided<br>*Only common tests should be described solely by name; describe more complex techniques in the Methods section.*
☒	☐	A description of all covariates tested
☐	☒	A description of any assumptions or corrections, such as tests of normality and adjustment for multiple comparisons
☐	☒	A full description of the statistical parameters including central tendency (e.g. means) or other basic estimates (e.g. regression coefficient) AND variation (e.g. standard deviation) or associated estimates of uncertainty (e.g. confidence intervals)
☐	☒	For null hypothesis testing, the test statistic (e.g. F, t, r) with confidence intervals, effect sizes, degrees of freedom and P value noted<br>*Give P values as exact values whenever suitable.*
☒	☐	For Bayesian analysis, information on the choice of priors and Markov chain Monte Carlo settings
☒	☐	For hierarchical and complex designs, identification of the appropriate level for tests and full reporting of outcomes
☒	☐	Estimates of effect sizes (e.g. Cohen's d, Pearson's r), indicating how they were calculated

Our web collection on statistics for biologists contains articles on many of the points above.

Software and code

Policy information about availability of computer code

Data collection	RT-qPCR were collected using QuantStudio 6 Pro Real-time PCR system by ThermoFisher (Applied Biosystems). Immunohistochemistry (IHC) sections were digitally scanned using the Hamamatsu Nanozoomer, Spectra Max i3X minimax imaging cytometer was utilized to quantify florescence, and lipid nanoparticle (LNP) characterization was performed with Zetasizer Nano ZS, BO LSRFortessa using BO FACSDiva v8.0.3 software. For the immunofluorescent (IF) assays, images were acquired on the 30 Histec Panoramic 250 scanner. For NanoString, samples were analyzed using nCounter digital analyzer. OC lipid concentration was determined via LC-MS analysis. A Waters Zevo TQs system equipped with a MRM detector was used for the chromatographic step.
Data analysis	Microsoft Excel (Office 365), Graphpad Prism v9, FlowJo vl0.7.1, and 30 Histec Caseviewer 2.4 (CaseViewer- 3DHISTECH Ltd.) to analyze fluorescent in situ hybridization (FISH) slides. QuPath was used to evaluate DLL3 IHC. Data collection was carried out on the nCounter Digital Analyzer (NanoString Technologies).

For manuscripts utilizing custom algorithms or software that are central to the research but not yet described in published literature, software must be made available to editors and reviewers. We strongly encourage code deposition in a community repository (e.g. GitHub). See the Nature Research guidelines for submitting code & software for further information.

Data

Policy information about availability of data

All manuscripts must include a data availability statement. This statement should provide the following information, where applicable:

- Accession codes, unique identifiers, or web links for publicly available datasets
- A list of figures that have associated raw data
- A description of any restrictions on data availability

The source data that support the findings of this study are provided with this paper.

Field-specific reporting

Please select the one below that is the best fit for your research. If you are not sure, read the appropriate sections before making your selection.

- [x] Life sciences
- [ ] Behavioural & social sciences
- [ ] Ecological, evolutionary & environmental sciences

For a reference copy of the document with all sections, see nature.com/documents/nr-reporting-summary-flat.pdf

Life sciences study design

All studies must disclose on these points even when the disclosure is negative.

Sample size	The sample size of each experiment was provided in the corresponding figure legend. Effect size between groups was predicted using historical data in corresponding tumor models (subcutaneous and orthotopic). Sample size for in vivo experiments was selected to ensure 90% confidence in assessment of a difference in tumor burden and statistical power analysis was performed with G*Power software (Faul, 2009).
Data exclusions	No animals and no data were excluded from the analysis.
Replication	All experiments were conducted in either duplicate or triplicate replication successfully.
Randomization	Animals were pair-matched based on tumor volume and randomly assigned to treatment arms.
Blinding	Blinding was not applicable for this nonclinical study.

Reporting for specific materials, systems and methods

We require information from authors about some types of materials, experimental systems and methods used in many studies. Here, indicate whether each material, system or method listed is relevant to your study. If you are not sure if a list item applies to your research, read the appropriate section before selecting a response.

Materials & experimental systems

n/a	Involved in the study	
[ ]	[x]	Antibodies
[ ]	[x]	Eukaryotic cell lines
[x]	[ ]	Palaeontology and archaeology
[ ]	[x]	Animals and other organisms
[x]	[ ]	Human research participants
[x]	[ ]	Clinical data
[x]	[ ]	Dual use research of concern

Methods

n/a	Involved in the study	
[x]	[ ]	ChIP-seq
[ ]	[x]	Flow cytometry
[x]	[ ]	MRI-based neuroimaging

Antibodies

Antibodies used	All antibodies were purchased from BioLegend (San Diego, USA), except CD69 Miltenyi Biotech (Bergisch Gladbach, Germany), mPD-1 antibody (Cat#: BE0146, Clone RMP1-14, Bio X Cell, Lebanon, NH), and rabbit antibody specific to human DLL3 (Clone E3J5R, Cell Signaling Technology, Danvers, MA).<br>The following BioLegend antibodies were used in the analysis: mouse CD45 (30-F11, Catalog # 103154), CD3 (epilson)(17A2, Catalog #100218), CD8a (53-6.7, Catalog # 100730), CD4 (RM4-5, Catalog # 100552), CD25 (PC61, Catalog # 102041), CD69 (REA937, Catalog #130-115-461), CTLA-4 (UC10-489, Catalog # 106323), NKP46 (29A1.4, Catalog # 137612), KLRG1 (2F1, Catalog # 138409), CD127 (A7R34, Catalog # 135035), PD-1 (29F.1A12, Catalog # 135216), MHCII (M5/114.15.2, Catalog # 107635), Ly6C (H, 1.4, Catalog # 128037), CD206 (C068C2, Catalog # 141720), CD86 (GL-1, Catalog # 105037), PD-L1 (10F.9G2, Catalog # 124312), and FOXP3 (150D, Catalog # 320008), DLL3 specific phycoerythrin-conjugated antibody (Catalog # 154003) and isotype control (Catalog # 400607).<br>IgG2a isotype control (clone 2A3, Catalog # BE0089, BioXCell, Lebanon, NH)<br>SVV rabbit polyclonal antisera was developed at Maine Biotechnology Services as described in this paper.
Validation	Each antibody was quality control tested according to its recommended usage as specified by the manufacturer. Validation can be found on the manufacturer's website. For the SVV rabbit polyclonal antisera, validation of neutralizing activity is presented in this paper.

Eukaryotic cell lines

Policy information about cell lines

Cell line source(s)	Cell lines NCI-H466 (HTB-171), NCI-H82 (HTB-175), NCI-H1299 (CRL-5803), SK-MEL-28 (HTB-72), CT26 (RL-2638), 4T1 (CRL-2539) and NIE-115 (CRL-2263) were all purchased from ATCC (Gaithersburg, MD). CT-2a-luc (SCC195) and MCC14/2

	(10092303) were purchased from Millipore Sigma (Burlington, MA). Murine colon adenocarcinoma cell line MC38 was kindly donated by Prof. Joseph Glorioso from the University of Pittsburgh.
Authentication	ATCC and Millipore Sigma provides commercially available certified and/or registered cell lines. Based on the manufacturer's website, ATCC offers a collection of products and services manufactured under ISO certification and accreditation. ATCC Standards Development Organization is accredited by the American National Standards Institute (ANSI) and develops standard protocols to ensure the reliability and reproducibility of biological materials. ASN-0002-Authentication of Human Cell Lines: Standardization of STR Profiling describes a consistent, inexpensive, and universally applicable method for authenticating new and established cell lines and their criteria for use. At Millipore Sigma, cell lines are authenticated by a CLEAR panel by Charles River Animal Diagnostic Services.
Mycoplasma contamination	All commercially available cell lines tested negative for mycoplasma contamination as confirmed by the manufacturers' websites (ATCC and Millipore Sigma). In particular, ATCC uses two methods of testing mycoplasma - the Hoechst DNA stain and direct culture method.
Commonly misidentified lines (See ICLAC register)	No commonly misidentified cell lines were used in the analysis.

Animals and other organisms

Policy information about studies involving animals; ARRIVE guidelines recommended for reporting animal research

Laboratory animals	In vivo experiments in xenograft tumor models NCI-H466, NCI-H82, NCI-H1299, and SK-MEL-28 were conducted in 8-12-week-old NU/NU nude female mice (Charles River Laboratories, Wilimington, MA). N1E-115 muring neuroblastoma tumor model was established in 8-12-week old A-J female mice (The Jackson Laboratory, Bar Harbor, ME). For experiments in 4T1 and CT26 tumor models, 8-12-week-old female BALB/c mice (Charles River Laboratories) were used. Studies using the MC38 and CT-2a-luc tumor model were conducted in 8-12-week-old female C57BL/6 mice (Charles River Laboratories). For experiments in the huICAM1 transgenic mice, 8-12-week-old mice were used (The Jackson Laboratory, Bar Harbor, ME). For experiments in the SCLC GEMM model, RMP mice 8 weeks of age were used (The Jackson Laboratory, Bar Harbor, ME). All animals had unlimited access to a sterile, pelleted rodent diet and reverse osmosis-purified water and were maintained on a 12:12-h light:dark cycle with access to environmental enrichment.
Wild animals	This study did not involve wild animals.
Field-collected samples	This study did not contain studies collected from the field.
Ethics oversight	All animal protocols were approved by the Oncorus Institutional Animal Care and Use Committee (IACUC) and were performed in accordance with IACUC regulations.

Note that full information on the approval of the study protocol must also be provided in the manuscript.

Flow Cytometry

Plots

Confirm that:

- [x] The axis labels state the marker and fluorochrome used (e.g. CD4-FITC).
- [x] The axis scales are clearly visible. Include numbers along axes only for bottom left plot of group (a 'group' is an analysis of identical markers).
- [x] All plots are contour plots with outliers or pseudocolor plots.
- [x] A numerical value for number of cells or percentage (with statistics) is provided.

Methodology

Sample preparation	As described in the Methods section in detail, briefly, tumors were weighed and then cut in small pieces before being disaggregated to single-cell suspensions by using the Tumor Dissociation Kit mouse (Catalog # 30-096-730, Miltenyi) and the Miltenyi GentleMACs Octo-dissociator with heaters according to the manufacturer's instructions.
Instrument	BD LSRFortessa Cell Analyzer
Software	Acquisition was performed using BD FACSDiva software v8.0.3 and analysis was performed using FlowJo_v10.7.1 software.
Cell population abundance	Sorting was not done; however, for FACS gating analysis cell [population of interest (ie., CD8) were collected as much as possible setting up a gate of acquisition of 50,000 cells.
Gating strategy	For the T cell and NK panel, cells were first gated for time (SSC-A vs Time), lymphocytes (SSC-A vs FSC-A) and singlets (FSC-H vs FSC-A). The lymphocyte gate was further analyzed for their uptake of the Live/Dead stain to determine live versus dead cells and CD45 expression. Then, the cells were gated on CD3 versus NKP46 to select T cells or NK cells. For the T cells, the population was then gated for CD4 versus CD8 and the CD4 T cells were further gated for CD25+ and FOXP3+ to analyze the Treg population. NK cells were gated for NKP46+ and CD3-. For the myeloid panel, cells were first gated for time (SSC-A vs Time) then gated for lymphocytes (SSC-A vs FSC-A) and singlets (FSC-H vs FSCA). The lymphocyte gate was further analyzed for their uptake of the Live/Dead stain to determine live versus dead cells and CD45 expression. Then, macrophages were

gated using SSC-A vs CD11b+ and subsequently on MHCII+Ly6C-subset. To distinguish between M1 and M2 macrophages, we used CD206-CD86+ for M1 and CD206+CD86- for M2.

☒ Tick this box to confirm that a figure exemplifying the gating strategy is provided in the Supplementary Information.
